# The genome sequence of a cave spider, *Troglohyphantes excavatus *Fage, 1919

**DOI:** 10.12688/wellcomeopenres.23920.1

**Published:** 2025-04-08

**Authors:** Martina Pavlek, Tin Rožman, Wellcome Sanger Institute Tree of Life Management, Samples and Laboratory team, Wellcome Sanger Institute Scientific Operations: Sequencing Operations, Wellcome Sanger Institute Tree of Life Core Informatics team, Tree of Life Core Informatics collective

**Affiliations:** 1 Ruder Boskovic Institute, Zagreb, Croatia; 2 Croatian Biospeleological Society, Zagreb, Croatia

**Keywords:** Troglohyphantes excavatus, cave spider, Dinaric karst, genome sequence, chromosomal, Araneae

## Abstract

We present a genome assembly from a female *Troglohyphantes excavatus* (cave spider; Arthropoda; Arachnida; Araneae; Linyphiidae). The genome sequence has a total length of 1,028.29 megabases. Most of the assembly (94.43%) is scaffolded into 13 chromosomal pseudomolecules. The mitochondrial genome has also been assembled, with a length of 14.82 kilobases.

## Species taxonomy

Eukaryota; Opisthokonta; Metazoa; Eumetazoa; Bilateria; Protostomia; Ecdysozoa; Panarthropoda; Arthropoda; Chelicerata; Arachnida; Araneae; Araneomorphae; Entelegynae; Orbiculariae; Araneoidea; Linyphiidae; Micronetinae; *Troglohyphantes*; *Troglohyphantes excavatus* Fage, 1919 (NCBI:txid1977180)

## Background

**Figure 1.**
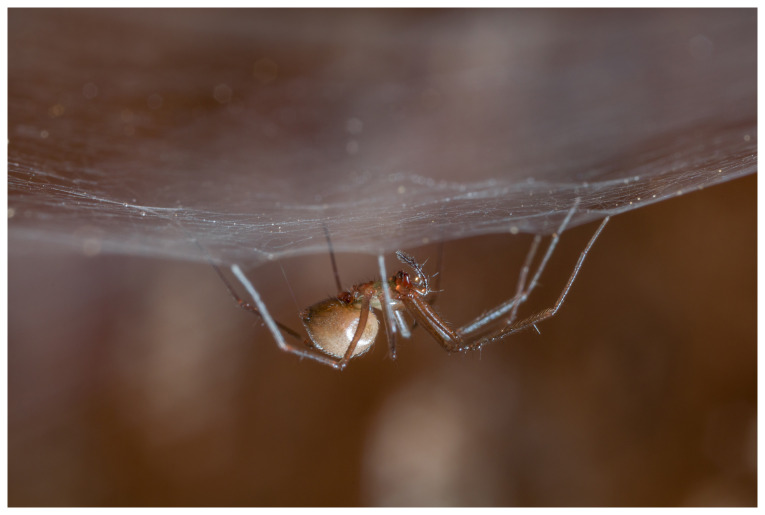
Photograph of *Troglohyphantes excavatus* from Donja Baraćeva špilja cave. Photo: Martina Pavlek.

The spider *Troglohyphantes excavatus* ( Figure 1) is a cave-dwelling spider distributed in south-east Europe. With a range of approximately 16,000 square kilometres, extending through Croatia, Slovenia, Italy, and Austria, it is one of the species with the largest distribution area in the genus *Troglohyphantes* ( Milano *et al.*, 2022). Although primarily found in caves, at its northern distribution border in Austria it is found in surface localities like in moss or in spruce or mixed forests ( Milano *et al.*, 2022; Thaler, 1986). Occasionally it can be found in surface habitats in the south of its distribution also, but always in moist and shadowed places ( Pavlek *et al.*, 2022). It is considered as troglophile ( Mammola *et al.*, 2022), meaning that although it is primarily a cave species, it can survive in certain surface habitats and use them for dispersal.

*Troglohyphantes excavates* is a relatively small spider with body size up to 3 mm, and leg length up to 9 mm ( Deeleman-Reinhold, 1978; Mammola *et al.*, 2022), brownish in colour, and with functional eyes. It spins a thin and gentle sheet web from which it hangs upside down. It preys on other small cave invertebrates like springtails, beetles, isopods and similar, or, when positioned near a cave entrance, on small flying insects that seek shelter in caves. The species is not listed on the IUCN red list, but its conservation status has been evaluated and is considered as least concern ( Milano *et al.*, 2022).

This is the first chromosome level genome of the genus *Troglohyphantes*. Most of its 138 species are found in caves, and they exhibit some level of cave adaptation, so this genome will be very important resource for studying evolution of cave adaptation in spiders and in invertebrates in general. Here we present the genome sequence of *Troglohyphantes excavatus*, based on a specimen from Špilja u kamenolomu Otruševac Cave, Otruševac Village.

## Genome sequence report

### Sequencing data

The genome of a specimen of *Troglohyphantes excavatus* was sequenced using Pacific Biosciences single-molecule HiFi long reads, generating 48.75 Gb from 3.96 million reads. GenomeScope analysis of the PacBio HiFi data estimated the haploid genome size at 839.34 Mb, with a heterozygosity of 1.08% and repeat content of 32.10%. These values provide an initial assessment of genome complexity and the challenges anticipated during assembly. Based on this estimated genome size, the sequencing data provided approximately 29.0x coverage of the genome. Chromosome conformation Hi-C sequencing produced 113.52 Gb from 751.79 million reads. Table 1 summarises the specimen and sequencing information.

**Table 1.** Specimen and sequencing data for *Troglohyphantes excavatus*.

Project information			
**Study title**	Troglohyphantes excavatus		
**Umbrella BioProject**	PRJEB71877		
**Species**	*Troglohyphantes excavatus*		
**BioSpecimen**	SAMEA14515608		
**NCBI taxonomy ID**	1977180		
Specimen information			
**Technology**	**ToLID**	**BioSample accession**	**Organism part**
**PacBio long read sequencing**	qqTroExca7	SAMEA14515645	whole organism
**Hi-C sequencing**	qqTroExca2	SAMEA10458248	whole organism
**RNA sequencing**	qqTroExca3	SAMEA10458249	whole organism
Sequencing information			
**Platform**	**Run accession**	**Read count**	**Base count (Gb)**
**Hi-C Illumina NovaSeq 6000**	ERR12507424	7.52e+08	113.52
**PacBio Sequel IIe**	ERR12510606	1.66e+06	18.7
**PacBio Sequel IIe**	ERR12510605	2.30e+06	30.04
**RNA Illumina NovaSeq 6000**	ERR12507425	7.20e+07	10.88

### Assembly statistics

The primary haplotype was assembled, and contigs corresponding to an alternate haplotype were also deposited in INSDC databases. The assembly was improved by manual curation, which corrected 58 misjoins or missing joins and removed 15 haplotypic duplications. These interventions reduced the total assembly length by 0.53%, decreased the scaffold count by 4.29%, and increased the scaffold N50 by 0.63%. The final assembly has a total length of 1,028.29 Mb in 133 scaffolds, with 464 gaps, and a scaffold N50 of 74.87 Mb ( Table 2).

**Table 2.** Genome assembly data for *Troglohyphantes excavatus*.

Genome assembly		
Assembly name	qqTroExca7.1	
Assembly accession	GCA_963932185.1	
*Alternate haplotype accession*	*GCA_963932215.1*	
Assembly level for primary assembly	chromosome	
Span (Mb)	1,028.29	
Number of contigs	597	
Number of scaffolds	133	
Longest scaffold (Mb)	82.79	
Assembly metric	Measure	*Benchmark*
Contig N50 length	3.43 Mb	*≥ 1 Mb*
Scaffold N50 length	74.87 Mb	*= chromosome N50*
Consensus quality (QV)	Primary: 57.8; alternate: 57.0; combined 57.4	*≥ 40*
*k*-mer completeness	Primary: 88.78%; alternate: 74.06%; combined: 98.45%	*≥ 95%*
BUSCO *	C:98.1%[S:93.1%,D:5.0%], F:0.7%,M:1.2%,n:2,934	*S > 90%; D < 5%*
Percentage of assembly mapped to chromosomes	94.25%	*≥ 90%*
Sex chromosomes	Not identified	*localised homologous pairs*
Organelles	Mitochondrial genome: 14.82 kb	*complete single alleles*

* BUSCO scores based on the arachnida_odb10 BUSCO set using version 5.5.0. C = complete [S = single copy, D = duplicated], F = fragmented, M = missing, n = number of orthologues in comparison.

**Figure 2.**
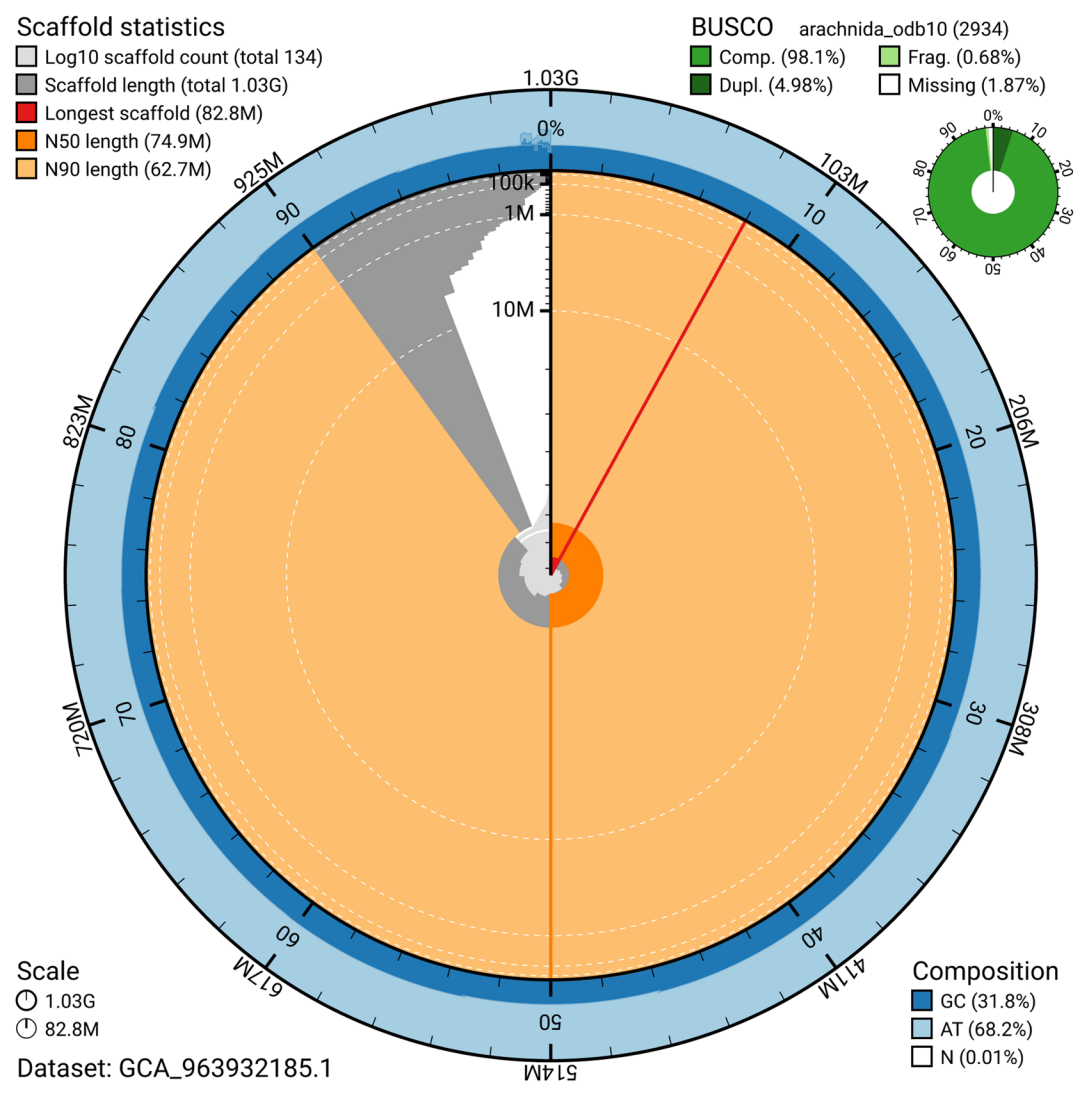
Genome assembly of *Troglohyphantes excavatus*, qqTroExca7.1: metrics. The BlobToolKit snail plot provides an overview of assembly metrics and BUSCO gene completeness. The circumference represents the length of the whole genome sequence, and the main plot is divided into 1,000 bins around the circumference. The outermost blue tracks display the distribution of GC, AT, and N percentages across the bins. Scaffolds are arranged clockwise from longest to shortest and are depicted in dark grey. The longest scaffold is indicated by the red arc, and the deeper orange and pale orange arcs represent the N50 and N90 lengths. A light grey spiral at the centre shows the cumulative scaffold count on a logarithmic scale. A summary of complete, fragmented, duplicated, and missing BUSCO genes in the arachnida_odb10 set is presented at the top right. An interactive version of this figure is available at https://blobtoolkit.genomehubs.org/view/GCA_963932185.1/dataset/GCA_963932185.1/snail.

**Figure 3.**
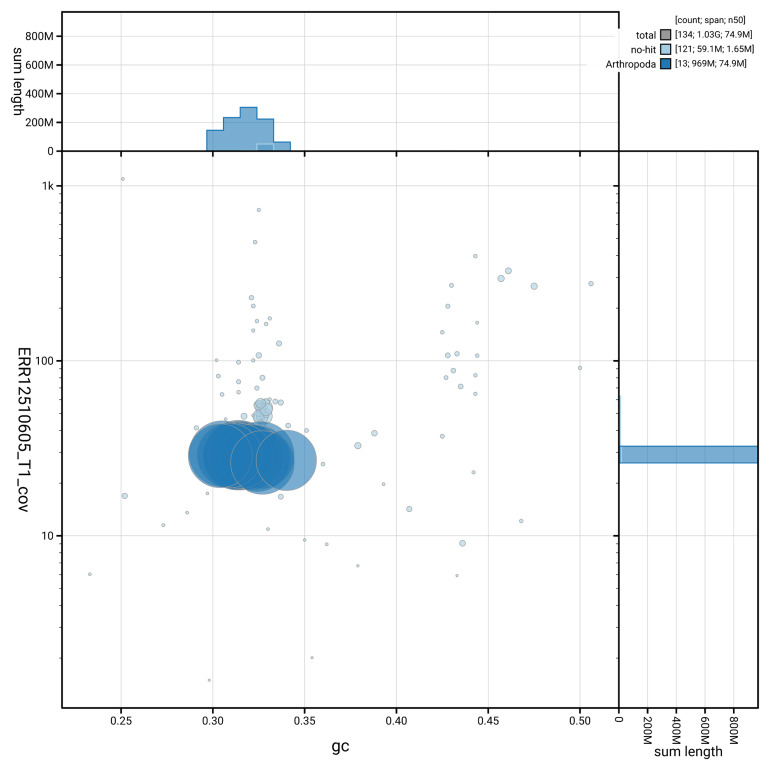
Genome assembly of *Troglohyphantes excavatus*, qqTroExca7.1: BlobToolKit GC-coverage plot. Blob plot showing sequence coverage (vertical axis) and GC content (horizontal axis). The circles represent scaffolds, with the size proportional to scaffold length and the colour representing phylum membership. The histograms along the axes display the total length of sequences distributed across different levels of coverage and GC content. An interactive version of this figure is available at https://blobtoolkit.genomehubs.org/view/GCA_963932185.1/dataset/GCA_963932185.1/blob.

**Figure 4.**
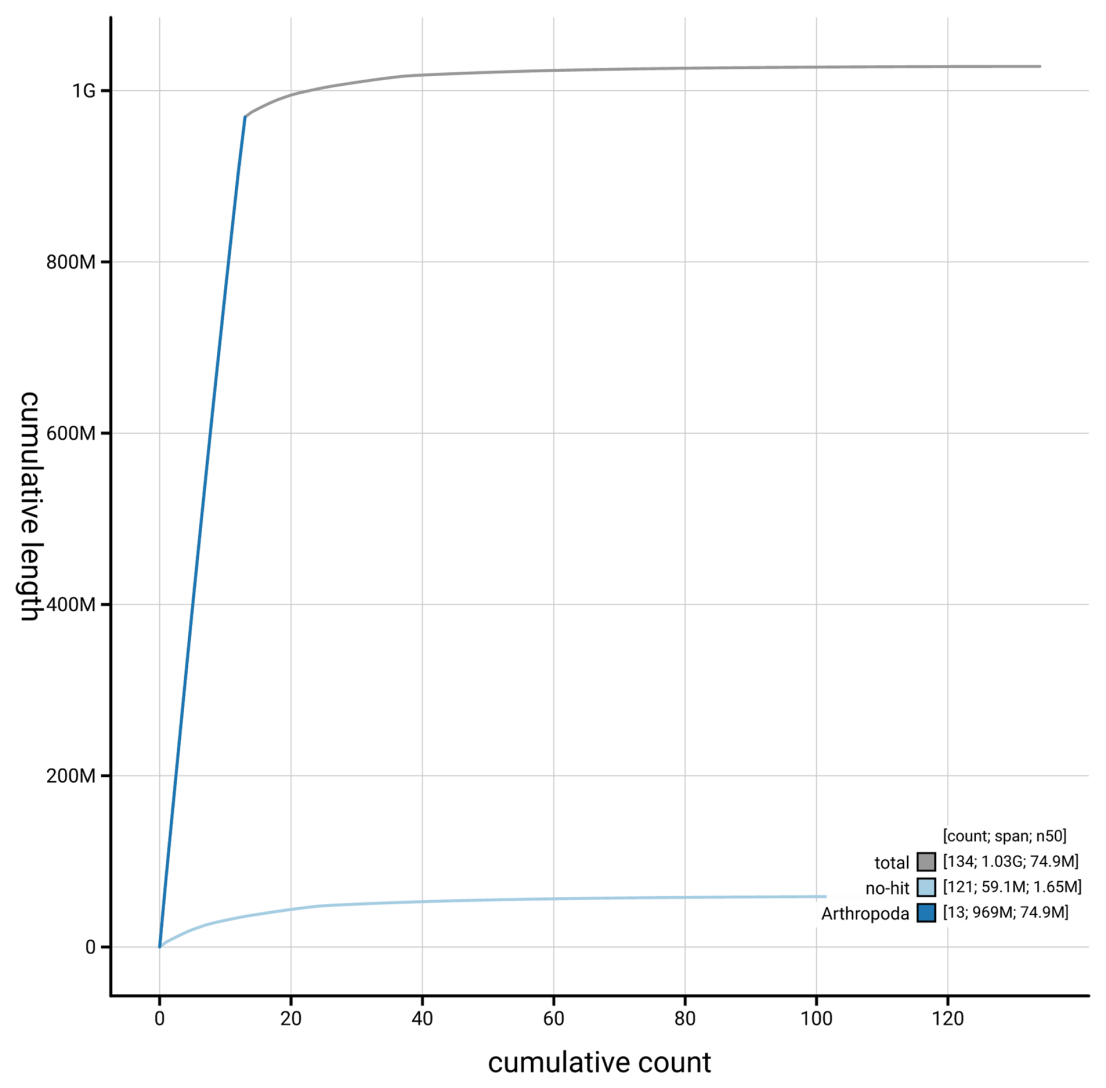
Genome assembly of *Troglohyphantes excavatus,* qqTroExca7.1: BlobToolKit cumulative sequence plot. The grey line shows cumulative length for all scaffolds. Coloured lines show cumulative lengths of scaffolds assigned to each phylum using the buscogenes taxrule. An interactive version of this figure is available at https://blobtoolkit.genomehubs.org/view/GCA_963932185.1/dataset/GCA_963932185.1/cumulative.

The snail plot in Figure 2 provides a summary of the assembly statistics, indicating the distribution of scaffold lengths and other assembly metrics. Figure 3 shows the distribution of scaffolds by GC proportion and coverage. Figure 4 presents a cumulative assembly plot, with separate curves representing different scaffold subsets assigned to various phyla, illustrating the completeness of the assembly.

**Figure 5.**
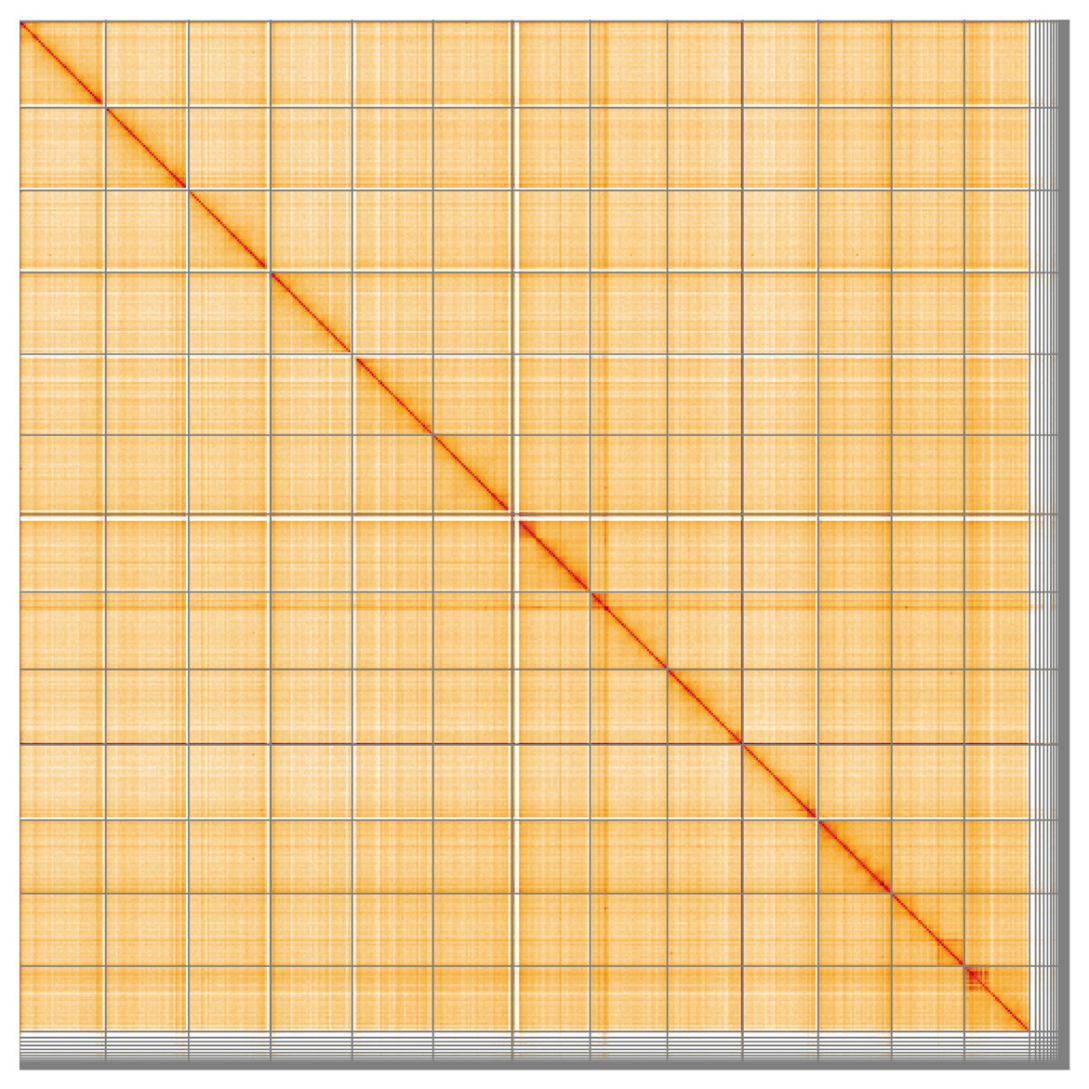
Genome assembly of *Troglohyphantes excavatus:* Hi-C contact map of the qqTroExca7.1 assembly, visualised using HiGlass. Chromosomes are shown in order of size from left to right and top to bottom. An interactive version of this figure may be viewed at https://genome-note-higlass.tol.sanger.ac.uk/l/?d=fl0fgFnaR8OaMMV4jWUjhg.

Most of the assembly sequence (94.25%) was assigned to 13 chromosomal-level scaffolds. These chromosome-level scaffolds, confirmed by Hi-C data, are named according to size ( Figure 5; Table 3). During curation, it was noted that the specimen is the homogametic sex (female), but no X chromosome could be identified.

**Table 3.** Chromosomal pseudomolecules in the genome assembly of *Troglohyphantes excavatus*, qqTroExca7.

INSDC accession	Name	Length (Mb)	GC%
OZ010666.1	1	82.79	31.5
OZ010667.1	2	79.46	31.5
OZ010668.1	3	78.79	31.5
OZ010669.1	4	78.27	31.5
OZ010670.1	5	77.59	32.5
OZ010671.1	6	75.81	32.5
OZ010672.1	7	74.87	30.5
OZ010673.1	8	74.11	32
OZ010674.1	9	72.31	31.5
OZ010675.1	10	72.27	31
OZ010676.1	11	70.42	30.5
OZ010677.1	12	69.77	32.5
OZ010678.1	13	62.71	34
OZ010679.1	MT	0.01	25

The mitochondrial genome was also assembled. This sequence is included as a contig in the multifasta file of the genome submission and as a standalone record.

### Assembly quality metrics

The estimated Quality Value (QV) and *k*-mer completeness metrics, along with BUSCO completeness scores, were calculated for each haplotype and the combined assembly. The QV reflects the base-level accuracy of the assembly, while *k*-mer completeness indicates the proportion of expected *k*-mers identified in the assembly. BUSCO scores provide a measure of completeness based on benchmarking universal single-copy orthologues.

The combined primary and alternate assemblies achieve an estimated QV of 57.4. The *k*-mer recovery for the primary haplotype is 88.78%, and for the alternate haplotype 74.06%; the combined primary and alternate assemblies have a *k*-mer recovery of 98.45%. BUSCO analysis using the arachnida_odb10 reference set ( *n* = 2,934) identified 98.1% of the expected gene set (single = 93.1%, duplicated = 5.0%).

Table 2 provides assembly metric benchmarks adapted from Rhie *et al.* (2021) and the Earth BioGenome Project (EBP) Report on Assembly Standards September 2024. The assembly achieves the EBP reference standard of **6.C.Q57.**

## Methods

### Sample acquisition

A female adult *Troglohyphantes excavatus* (specimen ID ERGA MP HR 06, ToLID qqTroExca7) was collected from Špilja u kamenolomu Otruševac cave, Otruševac Village (latitude 45.8199, longitude 15.6791) on 2022-03-26. The specimen was collected by Tin Rožman (Croatian Biospeleological Society, Zagreb, Croatia) and identified by Martina Pavlek (Ruder Boskovic Institute, Zagreb, Croatia).

The specimens used for Hi-C sequencing (specimen ID SAN0001794, ToLID qqTroExca2) and RNA sequencing (specimen ID SAN0001795, ToLID qqTroExca3) were collected from Donja Baraćeva špilja cave, Rakovica Village (latitude 44.9842, longitude 15.7232) on 2021-04-11. The specimens were collected and identified by Martina Pavlek (Ruder Boskovic Institute, Zagreb, Croatia).

### Nucleic acid extraction

The workflow for high molecular weight (HMW) DNA extraction at the Wellcome Sanger Institute (WSI) Tree of Life Core Laboratory includes a sequence of procedures: sample preparation and homogenisation, DNA extraction, fragmentation and purification. Detailed protocols are available on protocols.io ( Denton *et al.*, 2023b). The qqTroExca7 sample was prepared for DNA extraction by weighing and dissecting it on dry ice ( Jay *et al.*, 2023). Tissue from the whole organism was homogenised using a PowerMasher II tissue disruptor ( Denton *et al.*, 2023a). HMW DNA was extracted using the Automated MagAttract v2 protocol ( Oatley *et al.*, 2023a). DNA was sheared into an average fragment size of 12–20 kb in a Megaruptor 3 system ( Bates *et al.*, 2023). Sheared DNA was purified by solid-phase reversible immobilisation, using AMPure PB beads to eliminate shorter fragments and concentrate the DNA ( Oatley *et al.*, 2023b). The concentration of the sheared and purified DNA was assessed using a Nanodrop spectrophotometer and Qubit Fluorometer using the Qubit dsDNA High Sensitivity Assay kit. Fragment size distribution was evaluated by running the sample on the FemtoPulse system.

RNA was extracted from whole organism tissue of qqTroExca3 in the Tree of Life Laboratory at the WSI using the RNA Extraction: Automated MagMax™ *mir*Vana protocol ( do Amaral *et al.*, 2023). The RNA concentration was assessed using a Nanodrop spectrophotometer and a Qubit Fluorometer using the Qubit RNA Broad-Range Assay kit. Analysis of the integrity of the RNA was done using the Agilent RNA 6000 Pico Kit and Eukaryotic Total RNA assay.

### Hi-C sample preparation

Tissue from the whole organism of the qqTroExca2 sample was processed for Hi-C sequencing at the WSI Scientific Operations core, using the Arima-HiC v2 kit. In brief, 20–50 mg of frozen tissue (stored at –80 °C) was fixed, and the DNA crosslinked using a TC buffer with 22% formaldehyde concentration. After crosslinking, the tissue was homogenised using the Diagnocine Power Masher-II and BioMasher-II tubes and pestles. Following the Arima-HiC v2 kit manufacturer's instructions, crosslinked DNA was digested using a restriction enzyme master mix. The 5’-overhangs were filled in and labelled with biotinylated nucleotides and proximally ligated. An overnight incubation was carried out for enzymes to digest remaining proteins and for crosslinks to reverse. A clean up was performed with SPRIselect beads prior to library preparation. Additionally, the biotinylation percentage was estimated using the Qubit Fluorometer v4.0 (Thermo Fisher Scientific) and Qubit HS Assay Kit and Arima-HiC v2 QC beads.

### Library preparation and sequencing

Library preparation and sequencing were performed at the WSI Scientific Operations core.

***PacBio HiFi***

At a minimum, samples were required to have an average fragment size exceeding 8 kb and a total mass over 400 ng to proceed to the low input SMRTbell Prep Kit 3.0 protocol (Pacific Biosciences, California, USA), depending on genome size and sequencing depth required. Libraries were prepared using the SMRTbell Prep Kit 3.0 (Pacific Biosciences, California, USA) as per the manufacturer's instructions. The kit includes the reagents required for end repair/A-tailing, adapter ligation, post-ligation SMRTbell bead cleanup, and nuclease treatment. Following the manufacturer’s instructions, size selection and clean up was carried out using diluted AMPure PB beads (Pacific Biosciences, California, USA). DNA concentration was quantified using the Qubit Fluorometer v4.0 (Thermo Fisher Scientific) with Qubit 1X dsDNA HS assay kit and the final library fragment size analysis was carried out using the Agilent Femto Pulse Automated Pulsed Field CE Instrument (Agilent Technologies) and gDNA 55kb BAC analysis kit.

Samples were sequenced using the Sequel IIe system (Pacific Biosciences, California, USA). The concentration of the library loaded onto the Sequel IIe was in the range 40–135 pM. The SMRT link software, a PacBio web-based end-to-end workflow manager, was used to set-up and monitor the run, as well as perform primary and secondary analysis of the data upon completion.

***Hi-C***

For Hi-C library preparation, DNA was fragmented using the Covaris E220 sonicator (Covaris) and size selected using SPRISelect beads to 400 to 600 bp. The DNA was then enriched using the Arima-HiC v2 kit Enrichment beads. Using the NEBNext Ultra II DNA Library Prep Kit (New England Biolabs) for end repair, a-tailing, and adapter ligation. This uses a custom protocol which resembles the standard NEBNext Ultra II DNA Library Prep protocol but where library preparation occurs while DNA is bound to the Enrichment beads. For library amplification, 10 to 16 PCR cycles were required, determined by the sample biotinylation percentage. The Hi-C sequencing was performed using paired-end sequencing with a read length of 150 bp on an Illumina NovaSeq 6000 instrument.

***RNA***

Poly(A) RNA-Seq libraries were constructed using the NEB Ultra II RNA Library Prep kit, following the manufacturer’s instructions. RNA sequencing was performed on the Illumina NovaSeq 6000 instrument.

### Genome assembly, curation and evaluation

***Assembly***

Prior to assembly of the PacBio HiFi reads, a database of *k*-mer counts ( *k* = 31) was generated from the filtered reads using FastK. GenomeScope2 ( Ranallo-Benavidez *et al.*, 2020) was used to analyse the *k*-mer frequency distributions, providing estimates of genome size, heterozygosity, and repeat content.

The HiFi reads were assembled using Hifiasm ( Cheng *et al.*, 2021) with the --primary option. Haplotypic duplications were identified and removed using purge_dups ( Guan *et al.*, 2020). The Hi-C reads were mapped to the primary contigs using bwa-mem2 ( Vasimuddin *et al.*, 2019). The contigs were further scaffolded using the provided Hi-C data ( Rao *et al.*, 2014) in YaHS ( Zhou *et al.*, 2023) using the --break option for handling potential misassemblies. The scaffolded assemblies were evaluated using Gfastats ( Formenti *et al.*, 2022), BUSCO ( Manni *et al.*, 2021) and MERQURY.FK ( Rhie *et al.*, 2020).

The mitochondrial genome was assembled using MitoHiFi ( Uliano-Silva *et al.*, 2023), which runs MitoFinder ( Allio *et al.*, 2020) and uses these annotations to select the final mitochondrial contig and to ensure the general quality of the sequence.

***Assembly curation***

The assembly was decontaminated using the Assembly Screen for Cobionts and Contaminants (ASCC) pipeline. Flat files and maps used in curation were generated via the TreeVal pipeline ( Pointon *et al.*, 2023). Manual curation was conducted primarily in PretextView ( Harry, 2022) and HiGlass ( Kerpedjiev *et al.*, 2018), with additional insights provided by JBrowse2 ( Diesh *et al.*, 2023). Scaffolds were visually inspected and corrected as described by Howe *et al.* (2021). Any identified contamination, missed joins, and mis-joins were amended, and duplicate sequences were tagged and removed. The curation process is documented at https://gitlab.com/wtsi-grit/rapid-curation.

***Assembly quality assessment***

The Merqury.FK tool ( Rhie *et al.*, 2020), run in a Singularity container ( Kurtzer *et al.*, 2017), was used to evaluate *k*-mer completeness and assembly quality for the primary and alternate haplotypes using the *k*-mer databases ( *k* = 31) that were computed prior to genome assembly. The analysis outputs included assembly QV scores and completeness statistics.

A Hi-C contact map was produced for the final version of the assembly. The Hi-C reads were aligned using bwa-mem2 ( Vasimuddin *et al.*, 2019) and the alignment files were combined using SAMtools ( Danecek *et al.*, 2021). The Hi-C alignments were converted into a contact map using BEDTools ( Quinlan & Hall, 2010) and the Cooler tool suite ( Abdennur & Mirny, 2020). The contact map was visualised in HiGlass ( Kerpedjiev *et al.*, 2018).

The blobtoolkit pipeline is a Nextflow port of the previous Snakemake Blobtoolkit pipeline ( Challis *et al.*, 2020). It aligns the PacBio reads in SAMtools and minimap2 ( Li, 2018) and generates coverage tracks for regions of fixed size. In parallel, it queries the GoaT database ( Challis *et al.*, 2023) to identify all matching BUSCO lineages to run BUSCO ( Manni *et al.*, 2021). For the three domain-level BUSCO lineages, the pipeline aligns the BUSCO genes to the UniProt Reference Proteomes database ( Bateman *et al.*, 2023) with DIAMOND blastp ( Buchfink *et al.*, 2021). The genome is also divided into chunks according to the density of the BUSCO genes from the closest taxonomic lineage, and each chunk is aligned to the UniProt Reference Proteomes database using DIAMOND blastx. Genome sequences without a hit are chunked using seqtk and aligned to the NT database with blastn ( Altschul *et al.*, 1990). The blobtools suite combines all these outputs into a blobdir for visualisation.

The blobtoolkit pipeline was developed using nf-core tooling ( Ewels *et al.*, 2020) and MultiQC ( Ewels *et al.*, 2016), relying on the Conda package manager, the Bioconda initiative ( Grüning *et al.*, 2018), the Biocontainers infrastructure ( da Veiga Leprevost *et al.*, 2017), as well as the Docker ( Merkel, 2014) and Singularity ( Kurtzer *et al.*, 2017) containerisation solutions.

Table 4 contains a list of relevant software tool versions and sources.

**Table 4.** Software tools: versions and sources.

Software tool	Version	Source
BEDTools	2.30.0	https://github.com/arq5x/bedtools2
BLAST	2.14.0	ftp://ftp.ncbi.nlm.nih.gov/blast/executables/blast+/
BlobToolKit	4.3.9	https://github.com/blobtoolkit/blobtoolkit
BUSCO	5.5.0	https://gitlab.com/ezlab/busco
bwa-mem2	2.2.1	https://github.com/bwa-mem2/bwa-mem2
Cooler	0.8.11	https://github.com/open2c/cooler
DIAMOND	2.1.8	https://github.com/bbuchfink/diamond
fasta_windows	0.2.4	https://github.com/tolkit/fasta_windows
FastK	666652151335353eef2fcd58880bcef5bc2928e1	https://github.com/thegenemyers/FASTK
Gfastats	1.3.6	https://github.com/vgl-hub/gfastats
GoaT CLI	0.2.5	https://github.com/genomehubs/goat-cli
Hifiasm	0.19.8-r603	https://github.com/chhylp123/hifiasm
HiGlass	44086069ee7d4d3f6f3f0012569789ec138f42b84aa44357826c0b6753eb28de	https://github.com/higlass/higlass
MerquryFK	d00d98157618f4e8d1a9190026b19b471055b22e	https://github.com/thegenemyers/MERQURY.FK
Minimap2	2.24-r1122	https://github.com/lh3/minimap2
MitoHiFi	3	https://github.com/marcelauliano/MitoHiFi
MultiQC	1.14, 1.17, and 1.18	https://github.com/MultiQC/MultiQC
NCBI Datasets	15.12.0	https://github.com/ncbi/datasets
Nextflow	23.04.1	https://github.com/nextflow-io/nextflow
PretextView	0.2.5	https://github.com/sanger-tol/PretextView
purge_dups	1.2.5	https://github.com/dfguan/purge_dups
samtools	1.19.2	https://github.com/samtools/samtools
sanger-tol/ascc	-	https://github.com/sanger-tol/ascc
sanger-tol/blobtoolkit	0.5.1	https://github.com/sanger-tol/blobtoolkit
Seqtk	1.3	https://github.com/lh3/seqtk
Singularity	3.9.0	https://github.com/sylabs/singularity
TreeVal	1.2.0	https://github.com/sanger-tol/treeval
YaHS	1.2a.2	https://github.com/c-zhou/yahs

### Wellcome Sanger Institute – Legal and Governance

The materials that have contributed to this genome note have been supplied by a Tree of Life collaborator. The Wellcome Sanger Institute employs a process whereby due diligence is carried out proportionate to the nature of the materials themselves, and the circumstances under which they have been/are to be collected and provided for use. The purpose of this is to address and mitigate any potential legal and/or ethical implications of receipt and use of the materials as part of the research project, and to ensure that in doing so we align with best practice wherever possible.

The overarching areas of consideration are:

-    Ethical review of provenance and sourcing of the material
-    Legality of collection, transfer and use (national and international)

Each transfer of samples is undertaken according to a Research Collaboration Agreement or Material Transfer Agreement entered into by the Tree of Life collaborator, Genome Research Limited (operating as the Wellcome Sanger Institute) and in some circumstances other Tree of Life collaborators.

## Data Availability

European Nucleotide Archive: Troglohyphantes excavatus. Accession number PRJEB71877; https://identifiers.org/ena.embl/PRJEB71877. The genome sequence is released openly for reuse. The *Troglohyphantes excavatus* genome sequencing initiative is part of the European Reference Genome Atlas Pilot Project ( https://www.erga-biodiversity.eu/pilot-project). All raw sequence data and the assembly have been deposited in INSDC databases. The genome will be annotated using available RNA-Seq data and presented through the Ensembl pipeline at the European Bioinformatics Institute. Raw data and assembly accession identifiers are reported in Table 1 and Table 2.

## Author information

Members of the Wellcome Sanger Institute Tree of Life Management, Samples and Laboratory team are listed here: https://doi.org/10.5281/zenodo.12162482.

Members of Wellcome Sanger Institute Scientific Operations: Sequencing Operations are listed here: https://doi.org/10.5281/zenodo.12165051.

Members of the Wellcome Sanger Institute Tree of Life Core Informatics team are listed here: https://doi.org/10.5281/zenodo.12160324.

Members of the Tree of Life Core Informatics collective are listed here: https://doi.org/10.5281/zenodo.12205391.
