# Supplementary material for: The genome sequence of a cave spider, Troglohyphantes excavatus Fage, 1919
Source: Wellcome Open Res. 2025 Apr 8;10:177. [Version 1] doi: 10.12688/wellcomeopenres.23920.1 (PMC12485337; doi:10.12688/wellcomeopenres.23920.1)
Supplement: Peer review history

# Reviewer report: Reviewer response for version 1

**DOI:** 10.21956/wellcomeopenres.26389.r122437

**Published:** 2025-05-10

**Authors:** Ethan Briggs

**Affiliations:** 1 University of Idaho, Moscow, USA

**Reviewer report for “The genome sequence of a cave spider, *Troglohyphantes excavatus *Fage, 1919”**

 In this manuscript the authors assemble a *de novo* genome of a female *Troglohyphantes excavates*, using PacBio Hifi, HiC and RNAseq data. The background information on this species as well as the rationale for sequencing this spider is well explained. The protocols used for this study are appropriate and technically sounds, following the current, generally accepted protocols for genome assembly and assessment. The authors provide sufficient details to allow for replication. I have assumed that when a program is used, the default settings were implemented unless otherwise stated. Datasets in this study appear to be accessible, the accession numbers for the genome and raw data take the reader to the appropriate online depositories.

 A minor comment that I would like to see added to this paper, as well as other similar studies, is the citation of the paper the species was originally described. Not citing such papers overlooks the important work taxonomists provide. In this specific case, I ask the authors to cite: Fage 1919 Biospelogica XL. Etudes sur les araignées cavernicoles. III. Sur le genre *Troglohyphantes*. *Archives de Zoologie Expérimentale et Générale* 58: 55-148, pl. 2-8. 

 I recommend this manuscript be accepted and believe it will provide valuable genomic information for both arachnid and troglomorphic evolution.

Are sufficient details of methods and materials provided to allow replication by others?

Yes

Is the rationale for creating the dataset(s) clearly described?

Yes

Are the datasets clearly presented in a useable and accessible format?

Yes

Are the protocols appropriate and is the work technically sound?

Yes

Reviewer Expertise:

Genome assembly, phylogenomics, systematics, species delimitation

I confirm that I have read this submission and believe that I have an appropriate level of expertise to confirm that it is of an acceptable scientific standard.

## References

[1] Fage 1919 Biospelogica XL. Etudes sur les araignées cavernicoles. III. Sur le genre Troglohyphantes. Archives de Zoologie Expérimentale et Générale 58: 55-148, pl. 2-8.

# Reviewer report: Reviewer response for version 1

**DOI:** 10.21956/wellcomeopenres.26389.r122018

**Published:** 2025-05-07

**Authors:** Marc Domènech, Jesus Lozano-Fernandez

**Affiliations:** 1 Genètica, Microbiologia i Estadística, Universitat de Barcelona, Barcelona, Spain; 2 Universitat de Barcelona, Barcelona, Catalonia, Spain

This article provides the first chromosome-level genome for a spider in the genus *Troglohyphantes*. Although there are other genomes available for Araneae, even in the same family Linyphiidae, having the genome of a troglophile species can help study the genomic adaptations to life in caves.

 -In the second paragraph, "excavates" should be replaced by "excavatus".

 -In the third paragraph, according to the World Spider Catalog (as checked in 16 April 2025), there are 139 species of *Troglohyphantes*.

 -Although it would be optimal that all specimens used were collected in the same locality, especially when dealing with cave organisms, these species are so widespread in south-east Europe that it should not be a problem.

Are sufficient details of methods and materials provided to allow replication by others?

Yes

Is the rationale for creating the dataset(s) clearly described?

Yes

Are the datasets clearly presented in a useable and accessible format?

Yes

Are the protocols appropriate and is the work technically sound?

Yes

Reviewer Expertise:

Arachnology, phylogenomics

We confirm that we have read this submission and believe that we have an appropriate level of expertise to confirm that it is of an acceptable scientific standard.

# Reviewer report: Reviewer response for version 1

**DOI:** 10.21956/wellcomeopenres.26389.r122446

**Published:** 2025-04-28

**Authors:** Feng Zhang

**Affiliations:** 1 Nanjing Agricultural University, Nanjing, China

This Data Note effectively reports the generation of a high-quality, chromosome-level genome assembly for the cave spider *Troglohyphantes excavatus*. This represents the first such resource for this genus, which is relevant for studies of cave adaptation, speciation in fragmented habitats like the Dinaric Karst, and invertebrate evolution more broadly. The use of state-of-the-art sequencing (PacBio HiFi, Hi-C) and assembly methods, combined with thorough quality assessment (BUSCO, QV, k-mer completeness, Hi-C contact maps) and adherence to reporting standards (e.g., EBP standards), are major strengths. The data release is timely and valuable for the community. While the report is generally clear and comprehensive for its format.

**A minor comment:**

 The k-mer based estimate (GenomeScope) suggested a haploid size of ~839 Mb, while the final primary assembly is ~1.03 Gb. This ~23% difference is not insignificant and is not commented upon. Increase the parameter ‘-m’ ?

Are sufficient details of methods and materials provided to allow replication by others?

Yes

Is the rationale for creating the dataset(s) clearly described?

Yes

Are the datasets clearly presented in a useable and accessible format?

Yes

Are the protocols appropriate and is the work technically sound?

Yes

Reviewer Expertise:

genomics, phylogenomics

I confirm that I have read this submission and believe that I have an appropriate level of expertise to confirm that it is of an acceptable scientific standard.
